# Pulmonary paracoccidioidomycosis‐induced pulmonary hypertension

**DOI:** 10.1002/ctm2.213

**Published:** 2020-11-20

**Authors:** Sabrina Setembre Batah, Maiara Almeida Alda, Juliana Rodrigues Machado‐Rugulo, Renato Gonçalves Felix, Erika Nascimento, Roberto Martinez, Adriana Ignácio de Pádua, Eduardo Bagagli, Marluce Francisca Hrycyk, Hélio Cesar Salgado, Jaci Airton Castania, Lourenço Sbragia, Marcel Koenigkam Santos, José Antônio Baddini‐Martinez, Sirlei Siani Morais, Vera Luiza Capelozzi, Rosane Duarte Achcar, Alexandre Todorovic Fabro

**Affiliations:** ^1^ Department of Pathology and Legal Medicine Ribeirão Preto Medical School University of São Paulo Ribeirão Preto Brazil; ^2^ Institute of Biosciences, Botucatu Medical School São Paulo State University Botucatu Brazil; ^3^ Division of Infectious and Tropical Diseases Internal Medicine Department Ribeirão Preto Medical School University of São Paulo Ribeirão Preto Brazil; ^4^ Pulmonary Division Internal Medicine Department Ribeirão Preto Medical School University of São Paulo Ribeirão Preto Brazil; ^5^ Department of Physiology Ribeirão Preto Medical School University of São Paulo Ribeirão Preto Brazil; ^6^ Department of Medical Imaging, Hematology and Clinical Oncology Ribeirão Preto Medical School University of São Paulo Ribeirão Preto Brazil; ^7^ Department of Pathology Faculty of Medicine University of São Paulo São Paulo Brazil; ^8^ Department of Medicine Pathology Division National Jewish Health Denver Colorado USA

Dear Editor

Paraccoccidioidomycosis (PCM) is an endemic disease caused by the fungus *Paracoccidioidomycosis brasilensis*,^1^, ^2^ which when inhaled intensely and/or continuously reaches terminal bronchioles and alveoli promoting, in most of the cases, primary pulmonary paracoccidioidomycosis (PPCM).^2^, ^3^ While effective treatment is available to control PPCM, many patients considered clinically cured have developed pulmonary hypertension (PH). Yepez et al reported a detailed description of several PH cases in PPCM patients.^4^ Machado Filho et al showed the presence of late stage pulmonary hypertension post‐PCM infection in 23% of cases.^5^ Campos et al demonstrated that 24% of late stage post‐PCM infection patients had cor‐pulmonale, a final and advanced stage of PH.^6^ However, no additional clinical investigation is currently recommended to do PH screening.^2^ Therefore, we hypothesize that PPCM triggers PH, even after the infection is completely cured. As far as we know, our study is the first to demonstrate: (1) PCM limited to lung disease in animal model, (2) experimental PPCM‐induced PH demonstrated by right heart catheterization, and (3) progressive and aggressive phenotype of PH in PPCM patients.

To evaluate vascular involvement by PPCM‐infected subjects and subsequent role in pulmonary hypertension, fugal inoculation was performed by direct intrapulmonary needle puncture in male Wistar rats. The methodology detailed description can be found in the Supporting Information. After 8 weeks, the incidence rate of well‐formed granulomas in lung tissue was 60% (Figure 1A), with no evidence of PCM infection in other organs. Our methodology differs from other animal models of intramuscular, intraperitoneal, and specially intravenous, intranasal, and intratracheal instillation,^7^ which could infect other sites such as nasal cavity or esophagus by regurgitation or misplaced injection. While some areas of central necrosis were noted, the granulomas were largely nonnecrotizing, composed of epithelioid histiocytes and multinucleated giant cells, and seen in all lobes from both lungs in a predominantly peribronchioloar and miliary pattern of distribution.

**FIGURE 1:**
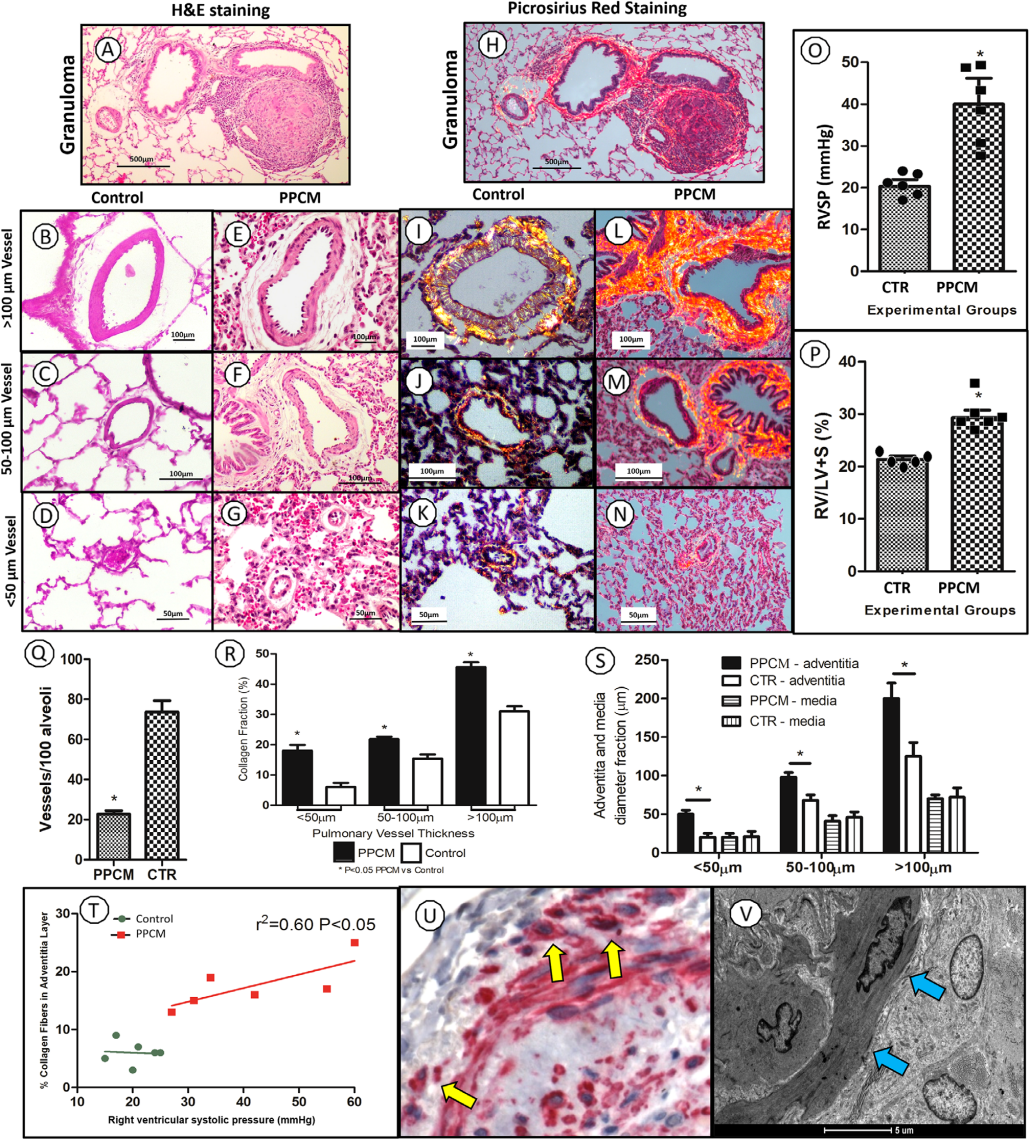
Experimental pulmonary paracoccidioidomycosis induced pulmonary hypertension. After intrapulmonary injection of 1 × 10^8^ paracoccidioidomycosis yeast, peribronchiolar well‐formed granulomas were seen (A). In contrast to controls (B, C, D), expanded adventitial layer of large (> 100 μm) (E), medium (50‐100 μm) (F), and small (<50 μm) (G) sized vessels were observed. Similarly, extracellular matrix deposition was present in peribronchiolar granulomas (H) and in all adventitial layer (L, M, N) compared to controls (I, J, K). RVSP was significantly higher in PCM subjects compared to the control group (O). Right ventricle to left ventricle plus septum (RV/LV+S) ratio was increased post‐PPCM infection, indicating increase in RV dilatation secondary to pulmonary hypertension (P). Precapillary vessels are decreased in PPCM compared to controls (Q). Collagen fraction is increased in all sizes of adventitial layer compared to controls (R) igual to adventitial diameter fraction, but not media (S). Perivascular collagen fibers deposition was directly correlated with an increase in mean pulmonary artery pressure in the PPCM group compared to the control group (*P* < .05) (T). Alfa smooth muscle actin (α‐SMA) immunoperoxidase stain (U) decorates vascular wall adventitial layer myofibroblastic activation (yellow arrows). This is further supported by transmission electron microscopy (V) identification of fibronexus (blue arrows) in adventitial layer. ^*^ *P* < .05

In contrast to control (Figure 1B–D), the arterial vessels showed expansion of adventitial collagen layer in all vessels (Figure 1E–G). In order to analyze extracellular matrix deposition in adventitial layers as the final result of myofibroblastic activation, the following methodologies were performed: picrossirius red staining, immunohistochemistry of alfa smooth muscle actin (α‐SMA), electron microscopy, and morphometry. Collagen fibers deposition was noted in perigranulomatous areas (Figure 1H) and large (> 100 μm)(Figure 1L), medium (50‐100 μm)(Figure 1M), and small (<50 μm)(Figure 1N) sized vessels compared to their controls (Figure 1I–K). Furthermore, the right ventricle systolic pressure (RVSP) was significantly higher in PPCM postinfection animals compared to control group (Figure 1O) with a corresponding increase in right ventricle (RV) to left ventricle plus septum (L+S) ratio, indicating RV dilatation secondary to PPCM‐induced PH (Figure 1P). To our knowledge, this is the first and unique study to catheterize the right ventricle and demonstrate experimental PPCM‐induced pulmonary hypertension. Moreover, a loss of small precapillary vessel (Figure 1Q), a common feature of precapillary pulmonary hypertension was observed. The adventitial layer collagen fibers’ deposition (Figure 1R) was significantly more pronounced in PPCM animals compared to the control group with increased adventitial diameter (Figure 1S), being directly correlated with right ventricular artery pressure (Figure 1T). The immunoexpression of α‐SMA (Figure 1U, yellow arrows) and identification of fibronexus by electron microscopy (Figure 1V, blue arrows) in the adventitial layer supports the hypothesis of adventitial myofibroblastic activation as a critical change in vascular remodeling. Our experimental data demonstrate the vascular adventitial layer remodeling and pulmonary hypertension induced in post‐PCM infection and suggest that in postcured PPCM patients there is a late self‐sustained myofibroblastic activation in the small vessels adventitia layer with a subsequent increase in perivascular collagen deposition and development of pulmonary hypertension, similar to histoplasmosis‐induced fibrosing mediastinitis and Idiopathic pulmonary fibrosis.^8^, ^9^

Fifteen lung biopsies from patients diagnosed with PCM between 2007 and 2017 were reviewed and showed similar findings to the animal model. Like in experimental data, significant expansion of the vascular adventitial layer by collagen fibers deposition was identified (Figure 2), which was more pronounced in small sized vessels (<50 μm) rather than medium‐ and large‐sized vessels, regardless the distance between the vessels and the granulomas. Vascular adventitial layer collagen fibers deposition was significantly increased in PCM patients compared to the chronic obstructive pulmonary disease (COPD)/emphysema group without PH (*P* < .05)(Figure 2), although COPD/emphysema is a known cause of vascular remodeling. Additional findings include some degree of peribronchial alveolar septal expansion by fibrosis (mild interstitial remodeling), alveolar septal rupture (emphysematous change), and intra‐alveolar macrophages with anthracotic pigment.

**FIGURE 2:**
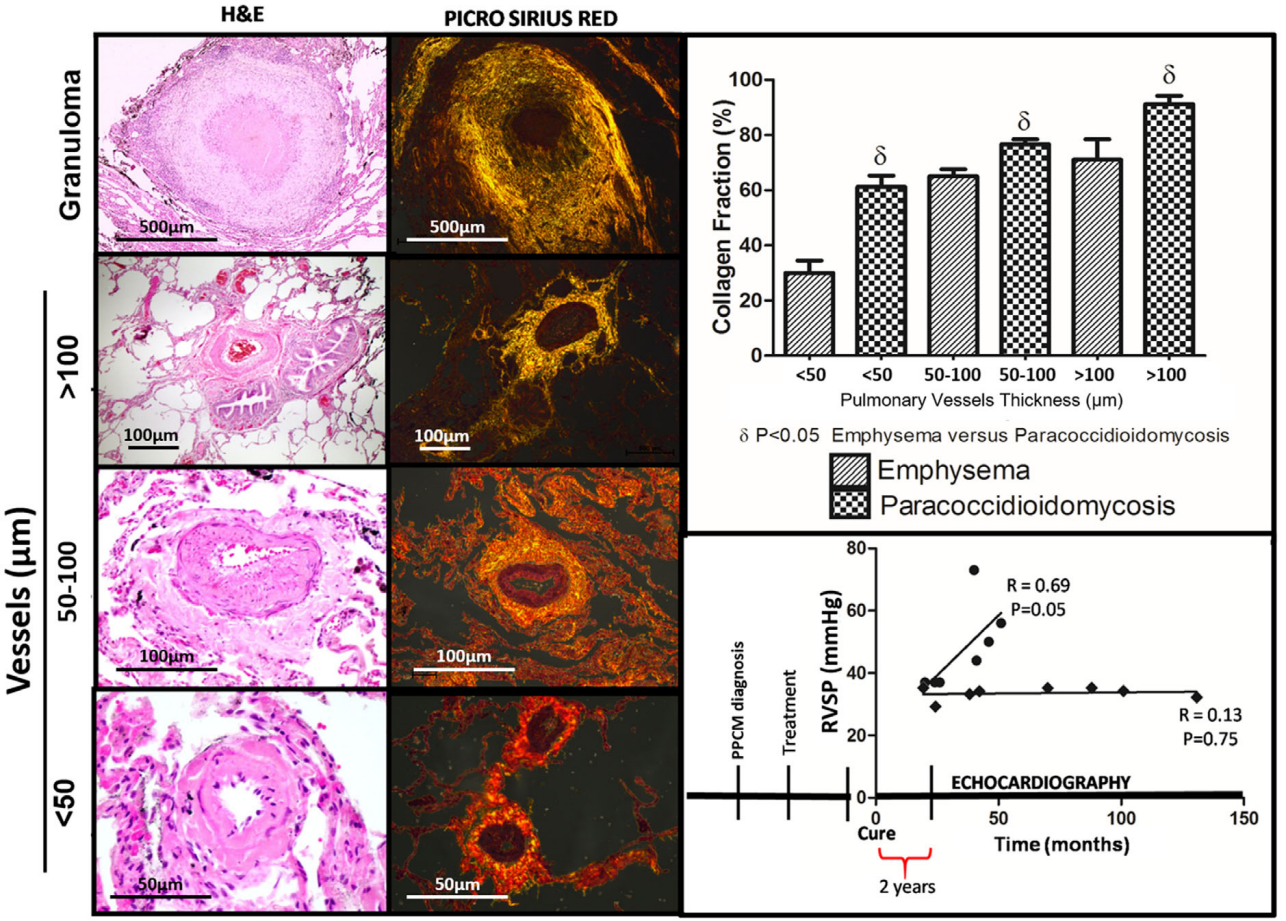
Human pulmonary paracoccidioidomycosis induced pulmonary hypertension. lung biopsy from PPCM patients and timeframe for development of pulmonary hypertension. Granulomas, expanded adventitial layer, and increased collagen fibers deposition in peri‐granuloma and perivascular areas were observed similar to animal model, highlighted by H&E and picrosirius red staining. Morphometrically, perivascular collagen fibers deposition was significantly increased in small (< 50 μm), medium (50‐100 μm), and large (>100 μm) sized vessels compared to the control group. Finally, two subsets of patients were established after PCM clinical cure (>2 years): (1) aggressive phenotype with progressive increase in RVSP; and (2) stable phenotype unassociated with progressive disease. δ *P* < .05

Retrospective clinical, radiological, echocardiographic (ECHO), and hemodynamic data were tabulated from electronic medical records of 510 patients considered clinically cured (Supporting Information and Table S1), according to Brazilian criteria guidelines during a 10‐year period (2007‐2017).^2^ Most of the patients were men between 50 and 60 years old and inveterate smokers. One third of the patients have COPD and a mean of 67 pack years of smoking history. Of 510 patients with pulmonary PCM, only 16 (3.14%) had echocardiography performed not as part of pulmonary hypertension work up but to investigate other possible suspected underlying cardiovascular condition, and none had right cardiac catheterization. Therefore, the diagnosis of pulmonary hypertension was based on ECHO, despite its limitations as a diagnostic method due to very high variability in terms of accurate pulmonary arterial pressure predictions. Of note, no underlying cardiovascular condition or history of tuberculosis was diagnosed in the study group/PCM population and no evidence of underlying interstitial lung disease or significant interstitial scarring was noted by high‐resolution computed tomography (Table S1). Half of the patients with echocardiography results recorded (*N* = 8) had RVSP higher than 35 mmHg after clinical cure of PPCM infection. While a RVSP cutoff of 35 mmHg resulted in 95% sensitivity for PH,^10^ a correlation between high RVSP and postinfection phase (Figure 2) indicated the presence of pulmonary hypertension in the postcure period (Table S1). The fact that the majority of PPCM patients enrolled in the study are smokers and COPD/emphysema can cause vascular remodeling, including pulmonary hypertension, can be considered a bias. However, RVSP in COPD does not usually exceed 30 mmHg and PH is not severe, unlike our findings (Figure 2).^11^

In conclusion, our experimental and human data indicate that PPCM‐induced pulmonary hypertension is an independent disease, triggered by post‐PCM infection in a substantial patient subset in late stage where clinical cure was achieved. The pathophysiologic mechanism seems to be linked to the adventitial layer of pulmonary vessels, a critical center for vascular remodeling mediated by proinflammatory and profibrotic microenvironment after PCM cure. While we acknowledge some limitations in our study such as ECHO for PH diagnosis and low number of PPCM patients with biopsies and ECHO, our goal is to not only encourage further investigation in a larger population with appropriate cardiology workup and long‐term follow‐up, but also eventually include this cause of pulmonary hypertension as a possibility in the clinical thinking process for diagnosis and management of late‐stage PPCM patients.

## ETHICAL APPROVAL

All human and experimental procedures were reviewed and approved by institutional review board, the Research Ethics Committee of Ribeirão Preto Medical School, University of São Paulo, Brazil, under the protocol 1.852.097.

## FUNDING

This work was supported by São Paulo Research Foundation [grant number: 17/23260‐9 and 16/23933‐0] and Brazilian Federal Agency for Support and Evaluation of Graduate) [grant number: 1807592‐2018].

## Supporting information

Supporting information

Supporting information
