# Supplementary material for: Pulmonary paracoccidioidomycosis‐induced pulmonary hypertension
Source: Clin Transl Med. 2020 Nov 20;10(7):e213. doi: 10.1002/ctm2.213 (PMC7678439; doi:10.1002/ctm2.213)
Supplement: Supplementary file 1 — Supporting information [file CTM2-10-e213-s001.docx]

**Methods**

***Ethics statement***

All human and animal experiments complied with the human and animal experiment regulations and Ethical Guidelines of the National Institutes of Health. All human and experimental procedures were reviewed and approved by institutional review board, the Research Ethics Committee of Ribeirão Preto Medical School, University of São Paulo, Brazil under the protocol 1.852.097.

***Animals and experimental treatment***

Male Wistar rats were randomized into two groups: paracoccidioides brasiliensis-induced group (Pb group, N=6) and control group (N=6). The animals received intraperitoneal administration of anesthesia with 0.3mg / kg of xylazine (Rompum, Bayer, Brazil) and 10mg / kg ketamine hydrochloride (Ketalar, Park-Davis). Each entire lung received a single intrapulmonary injection of 1x10^8^ paracoccidioidomycosis yeast diluted in 500 microliters saline solution. The solution was injected with a 22-gauge needle at a 30-degree angle between the 2nd and 3rd rib in the mid- clavicular line. After deep insertion of the needle by 2 cm, the lung parenchyma (right middle lobe and lingula respectively) was reached. After that, the syringe plunger was pulled back and air filled (0.1 mL) and the 500 microliters solution was then slowly injected while the needle was slowly and progressively removed from the lung. The control group received saline solution. Both groups were provided with a standard diet *ad libitum*, housed in cages and exposed to a light and dark cycle of 12 hours. After 8 weeks, rats from both groups were sedated and anesthetized (10 mg/kg intraperitoneal ketamine from Park-Davisand 0.3 mg/kg, and xylazine from Rompum, Bayer, Brazil) for right heart catheterization, followed by euthanasia. Tracheal dissection was performed, followed by right and left pulmonary lobes, removing the lungs. A Silastic cannula was introduced into the trachea for saline infusion for 10 minutes. Under 25mmHg pressure, the right lung was fixed with 10% buffered formalin and the left lung with 10 mL of 4% paraformoldehyde for 2h.  The lung was paraffin embedded and sampled using the standards for quantitative assessment of pulmonary structures by ATS/ERS[^1^](#_ENREF_1).

***Patients and biopsy***

The cases were selected from patients diagnosed with paracoccidioidomycosis from 2007 to 2017 at Clinic Hospital of Ribeirão Preto Medical School, University of São Paulo, Brazil, and the clinical, radiologic and echocardiographic data were collected from respective electronic medical records (Table-S1). Fifteen transbronchial biopsies from PPCM patients (study group) and 5 COPD/emphysema autopsy cases with documented normal echocardiogram (control group) were obtained from pathology service and reviewed by 2 pulmonary pathologists’ authors (ATF and VLC) blinded for clinical history. The tissue from transbronchial biopsies and autopsy cases were sampled from lung peribronchial/centrilobular zones. Control group and study group were matched by gender, age and smoking history.

***Right heart catheterization***

Hemodynamic measurements were performed with a 3.5F pressure transducer catheter (Millar Instruments) and analyzed by AcqKnowledge software (Biopac Systems Inc.) as previously described[^2^](#_ENREF_2). The formalin fixed heart was dissected to perform appropriate measurements such as right ventricle (RV) to left ventricle plus septum (LV+S) ratio.

***Histochemistry and histomorphometry***

Human and experimental lung tissue were cut in 3-mm sections and stained with hematoxylin and eosin (H&E) and picrosirius red stain. The total amount of collagen deposition present in adventitial vascular and perivascular layer and adjacent interstitial was quantified by images acquired by Novel trinocular microscope (L3000 super LED). Morphometry analysis were performed with Image-Pro Plus software, according to our laboratory standard protocol, as described in detail by Fabro et al[^3^](#_ENREF_3), and according to ATS/ERS morphometry guidelines[^1^](#_ENREF_1). Briefly, small arterioles, medium and large sized pulmonary arteries were divided into 3 sub-groups determined by their minor external diameter: <50um (small sized arterioles), 50-100um (medium sized arteries), and >100um (large sized arteries)[^4^](#_ENREF_4). Ten representative small, medium and large sized pulmonary arteries from each subject (including animal and human model) were selected and perivascular collagen thickness measured in high power field (400X). Additionally, in order to investigate the loss of small precapillary vessel in PPCM animal e human group, the quantification of precapillary vessel per 100 alveoli were performed by 10 images acquired by Novel trinocular microscope (L3000 super LED).

***Immunohistochemistry***

A standard peroxidase technique with biotinylated rabbit polyclonal antibody anti-α-SMA+ cells (Ab ab5694) was performed at 1:100 dilution. The Max Polymer Novolink amplification kit (Leica, Newcastle Inc., UK) was carried out for signal amplification and 3,3′-diaminobenzidine tetrachloride was used as a precipitating substrate for signal detection.

***Electron microscopy***

Ultrastructural electron microscopy was performed using ultrathin rat lung sections fixed in 4% paraformaldehyde and embedded in Lowicryl HM-20 resin, following the protocol established by our laboratory[^3^](#_ENREF_3).

***Statistical analysis***

The data were expressed as the mean ± standard deviation. All statistical procedures were performed using SPSS software v.13.0(SPSS, Inc., Chicago, IL, 2004). The data were assessed using the t test and ANOVA with Tukey or Dunnett post hoc tests for multiple comparisons when the data were normal and the variances were homogeneous. Pearson correlation was used for assessment of the relationships among variables. A p value of less than 0.05 was considered statistically significant.

**References**

1. Hsia CC, Hyde DM, Ochs M, Weibel ER, Structure AEJTFoQAoL. An official research policy statement of the American Thoracic Society/European Respiratory Society: standards for quantitative assessment of lung structure. *Am J Respir Crit Care Med.* Feb 15 2010;181(4):394-418.

2. Dai Z, Zhu MM, Peng Y, et al. Endothelial and Smooth Muscle Cell Interaction via FoxM1 Signaling Mediates Vascular Remodeling and Pulmonary Hypertension. *Am J Respir Crit Care Med.* Sep 15 2018;198(6):788-802.

3. Fabro AT, da Silva PH, Zocolaro WS, et al. The Th17 pathway in the peripheral lung microenvironment interacts with expression of collagen V in the late state of experimental pulmonary fibrosis. *Immunobiology.* Jan 2015;220(1):124-135.

4. Arnold ND, Pickworth JA, West LE, et al. A therapeutic antibody targeting osteoprotegerin attenuates severe experimental pulmonary arterial hypertension. *Nature communications.* Nov 15 2019;10(1):5183.
