# Supplementary material for: Pulmonary paracoccidioidomycosis‐induced pulmonary hypertension
Source: Clin Transl Med. 2020 Nov 20;10(7):e213. doi: 10.1002/ctm2.213 (PMC7678439; doi:10.1002/ctm2.213)
Supplement: Supplementary file 2 — Supporting information [file CTM2-10-e213-s002.docx]

**Table S1 - Clinical, tomographic and echocardiographic parameters of PPCM patients** categorized by normal (≤35) or increased (>35) RVSP (mmHg).

| **PARAMETERS** | | **RVSP (mmHg)** | | **P value** |
| --- | --- | --- | --- | --- |
|  |  | **≤35**  **(N=8)** | **>35**  **(N=8)** |  |
| **CLINICAL** | **Gender (M:F)** | 7:1 | 6:2 | **-** |
|  | **Age (yrs)*** | 49.50 ± 3.16 | 51.50 ± 4.81 | 0.73 |
|  | **Cough** | 2 | 5 | 0.16 |
|  | **Dyspnea** | 2 | 5 | 0.16 |
|  | **Comorbidities** | 4 | 4 | 0.95 |
|  | **COPD** | 3 | 5 | 0.63 |
|  | **Smoking** | 5 | 6 | 0.65 |
|  | **Pack-year*** | 59 ± 9.00 | 76.67 ± 21.86 | 0.59 |
| **PFT** | **FEV1*** | 1.9 ± 0.08 | 1.69 ± 0.1 | 0.16 |
|  | **FVC*** | 3,5 ± 0,06 | 3.4 ± 0.04 | 0.12 |
|  | **FEV1/FVC*** | 0.53 ± 0.02 | 0.54 ± 0.03 | 0.96 |
|  | **FEF 25–75*** | 0.81 ± 0.03 | 0.79 ± 0.04 | 0.74 |
| **TOMOGRAPHIC** | **PA d (mm)*** | 25.33 ± 1.25 | 29.57 ± 2.20 | 0.14 |
|  | **AAo d (mm)*** | 32.83 ± 2.02 | 32.86 ± 1.68 | 0.99 |
|  | **PA / AAo*** | 0.77 ± 0.02 | 0.919 ± 0.087 | 0.17 |
|  | **RV/LV (mm)*** | 0.83 ± 0.08 | 0.98 ± 0.14 | 0.40 |
|  | **SA/B (S1D-mm)*** | 1.11 ± 0.07 | 1.08 ± 0.09 | 0.80 |
|  | **Opacities** | 5 | 6 | 0.65 |
|  | **Emphysema** | 1 | 4 | 0.13 |
|  | **Cardiomegaly** | 0 | 2 | - |
| **ECHOCARDIOGRAPHIC** | **Height (cm)*** | 168.1 ± 3.22 | 162.4 ± 3.34 | 0.24 |
|  | **Weight (kg)*** | 78.76 ± 10.34 | 56.63 ± 2.08 | 0.06 |
|  | **BSA (m²)*** | 1.74 ± 0.11 | 1.59 ± 0.03 | 0.22 |
|  | **EDD (cm)*** | 47.81 ± 1.62 | 45.08 ± 1.79 | 0.28 |
|  | **ESD (cm)*** | 28.14 ± 0.83 | 27.60 ± 1.96 | 0.78 |
|  | **FS (%)*** | 40.67 ± 0.76 | 38.70 ± 1.50 | 0.13 |
|  | **EF (%)*** | 67.75 ± 3.10 | 61.38 ± 4.43 | 0.26 |
|  | **TF *** | 46.75 ± 2.38 | 53.57 ± 4.76 | 0.21 |
|  | **PVF*** | 74.13 ± 2.95 | 71.50 ± 5.57 | 0.68 |
|  | **HR (bpm)*** | 75.75 ± 4.79 | 83.67 ± 5.12 | 0.29 |
|  | [**RVSP**](https://www.ncbi.nlm.nih.gov/pmc/articles/PMC5454185/#s0005title)***** | 33.38 ± 0.73 | 46.38 ± 4.57 | 0.010 |

**Note:** *mean ± standard deviation. **Abbreviations:** M, male; F, female; COPD, chronic obstructive pulmonary disease; PFT, pulmonary function test; FEV1, Forced expiratory volume at timed interval of 1.0 seconds; FVC, Forced vital capacity; FEF 25–75, forced expiratory flow 25–75%; BSA, body surface area; EDD, end-diastolic diameter; ESD, end-systolic diameter; d, diameter; FS, fractional shortening; EF, ejection fraction; HR, heart rate; TF, tricuspid flow; PVF, pulmonary venous flow; RVSP, right ventricular systolic pressure; PA, pulmonary artery; AAo, ascending aorta; RV, right ventricular wall thickness (mm); LV, left ventricular wall thickness (mm); SA, segmental arteries; B, bronchi.
